# Insect recognition based on complementary features from multiple views

**DOI:** 10.1038/s41598-023-29600-1

**Published:** 2023-02-20

**Authors:** Jingmin An, Yong Du, Peng Hong, Lei Zhang, Xiaogang Weng

**Affiliations:** 1 School of Life Sciences, Northeast Agricultural University, Harbin, China; 2 State Key Laboratory of Membrane Biology, Institute of Zoology, Chinese Academy of Sciences, Beijing, China; 3 College of Intelligence and Computing, Tianjin University, Tianjin, China; 4 School of Electrical and Information Engineering, Northeast Agricultural University, Harbin, China; 5 Software College, Northeastern University, Shenyang, China; 6 Neusoft Research of Intelligent Healthcare Technology, Co. Ltd., Shenyang, China; 7 Department of Diagnostic Radiology and Nuclear Medicine, University of Maryland School of Medicine, Baltimore, MD USA

**Keywords:** Computer science, Image processing

## Abstract

Insect pest recognition has always been a significant branch of agriculture and ecology. The slight variance among different kinds of insects in appearance makes it hard for human experts to recognize. It is increasingly imperative to finely recognize specific insects by employing machine learning methods. In this study, we proposed a feature fusion network to synthesize feature presentations in different backbone models. Firstly, we employed one CNN-based backbone ResNet, and two attention-based backbones Vision Transformer and Swin Transformer to localize the important regions of insect images with Grad-CAM. During this process, we designed new architectures for these two Transformers to enable Grad-CAM to be applicable in such attention-based models. Then we further proposed an attention-selection mechanism to reconstruct the attention area by delicately integrating the important regions, enabling these partial but key expressions to complement each other. We only need part of the image scope that represents the most crucial decision-making information for insect recognition. We randomly selected 20 species of insects from the IP102 dataset and then adopted all 102 kinds of insects to test the classification performance. Experimental results show that the proposed approach outperforms other advanced CNN-based models. More importantly, our attention-selection mechanism demonstrates good robustness to augmented images.

## Introduction

Insect pests, which severely hinder the development of kinds of plants, are particularly harmful to the ecological environment. Accurate insect recognition is increasingly important for warning the upcoming insect disasters and an effective way to prevent large-scale intrusion. The variance in morphology among different types of insects is relatively slight, and the same insect species hold different stages, including egg, larva, pupa and adult^1^. In other words, significant intra-class differences and large inter-species similarities make the recognition of insect pests challenging.

Convolutional Neural Networks (CNN) have demonstrated remarkable image classification potential^2^, which has also been widely applied for classifying insect images. Wang et al. designed a CRAFNet model which introduced VGG-a and Inception modules, and reached 92.26% recognition accuracy in their constructed insects dataset named CRAF^3^. Liu et al. constructed a Deep Feature Fusion Residual Network (DFF-ResNet) by stacking fused features from a previous layer between two 1 × 1 convolution layers in a residual signal branch based on the original ResNet^4^. Li et al.^5^ finetuned GoogLeNet to deal with the complicated backgrounds of farmland scenes, with pest classification accuracy 6.2% better than ResNet101. Nanni et al. proposed an automatic classifier based on the fusion between three different saliency methods and five convolutional neural networks^6^. They explored the possibility of combining CNNs and saliency methods to create an ensemble of classifiers combined by the sum rule. Cheng et al.^7^ used deep residual learning in complex farmland backgrounds and got 98.67% classification accuracy for ten classes of crop pest images.

Many pest datasets are also widely studied, in which the classes of insects largely vary. Wang et al. established an image database including 19 insects and one larva^3^. Li et al. collected 5629 images of 10 species of crop pests by downloading and crawling from popular search engines^5^. A recently published IP41 dataset compromises 46,567 original images of crop pests in 41 classes^8^. We used the IP102 dataset^9^, a highly challenging public dataset including 102 kinds of insects, where almost every pest category includes more than one growth form (i.e., pupa, larva, adult). Many advanced studies have been implemented on this dataset, which obtained improved performances in varying degrees^6,10,11^ or less computation time^12^. It is worth mentioning that Gomes et al. curated the images from IP102 to compromise two datasets composed of adult insect and early-stage insect images^13^. They got significantly higher accuracy than the work implemented on the original IP102 dataset that mixed multiple growth stages in a specific class, demonstrating that different growth forms indeed increase the difficulty of recognition.

Discriminative localization is essential for fine-grained image classification tasks^14^, and prior studies showed that using local region information helps to improve recognition performance^15^. Prior studies have utilized localized, well-defined features to facilitate downstream classifications. For example, He et al.^14^ proposed a discriminative localization method via saliency-guided Faster R-CNN. The saliency information was extracted by a saliency extraction network, providing the bounding box for training fast R-CNN to localize discriminative regions and identify specific birds. Additionally, Zhang et al. proposed a three-step image emotion recognition pipeline that leverages emotion intensity learning^15^. Specifically, they adopted class activation mapping techniques to generate pseudo intensity maps for emotion intensity learning, and the predicted intensity map is introduced to the final classification network for emotion recognition. Gradient-weighted Class Activation Mapping (Grad-CAM) is a noteworthy study concerning localizing discriminative features. It uses the gradient of any target category and flows into the final convolutional layer to produce a coarse localization map that highlights the important regions^16^. It has been widely used in image classification, image captioning and visual question-answering models. Afterward, various enhanced variants of Grad-CAM were designed. Grad-CAM +  + ^17^ outperformed Grad-CAM when explaining the occurrence of multiple object instances in a single image. Score-CAM^18^ gets rid of the dependence on gradients by obtaining the weight of each activation map through its forward passing score on the target class. Finally, a linear combination of weights and activation maps is used to get the final results. Ablation CAM^19^ uses ablation analysis to determine the importance (weights) of individual feature map units with respect to class. Eigen-CAM^20^ computes and visualizes the principle components of the learned representations from the convolutional layers. We conducted rigorous test among all the mentioned variants and Grad-CAM itself, and the original Grad-CAM by^11^ obtained the highest Intersection-over-Union (IOU) between the true segmentation label and the highlighted regions under 264 manually-labeled insect samples. In other words, Grad-CAM can better localize the region of the insect body in the image data we used. In the following chapter, we use CAMs (Class Activation Maps) to refer to the mapping generated by any model with Grad-CAM.

Many studies have implemented Grad-CAM to show the effectiveness and interpretability of their CNN-based models in various vision applications. One of the potential scenarios is making deep models more explainable, especially in medical areas^21^. In recent years, Grad-CAM has been widely applied to facilitate interpreting how the machines get the specific lesion areas, such as the detection of COVID-19 on chest CT^22,23^. Prior researchers also employed Grad-CAM to see whether their proposed deep models accurately captured insect regions^24–26^. Compared to ResNet50, for example, a proposed Deep Multi-branch Fusion Residual Network (DMF-ResNet) obtained wider and more precise highlight regions for the same insect image^24^. It is worth noticing that Yang et al. employed the key regions with the largest discriminative features for fine-grained insect classification^27^. However, their key area features were generated by one single model ResNet, which did not synthesize multi-view features. In fact, different models can extract complementary features, and delicately fusing them can induce better recognition performance. In essence, Grad-CAM illustrates the exact key area feature extracted from the corresponding model. Therefore, the highlighted key areas depicted by different backbones vary. Based on these important features provided by multiple models, we proposed a feature fusion framework that delicately combines their focused regions. This method comprehensively considers inherent characteristics in different architectures and brings in diversified and complementary features.

The attention mechanism originates from Transformer^28^, and it is first proposed to deal with natural language sequences. It has become a popular and well-performed approach in vision domains, in which an image is usually split into small patches as the input. Unlike the CNN-based model that focuses on extracting local features, attention-based architectures tend to find representative global information and the intrinsic relation in the structure. The attention information in these separated patches can be discrepant that provide equally important references. However, few studies employed Transformers for insect recognition tasks. More importantly, taking advantage of both the feature-extraction ability of CNN and the Transformer is seldom discussed. The two types of architecture capture complementary information in one image, and delicately combining this information may improve insect recognition performance. Therefore, the attention-selection mechanism is proposed to fuse these features to enable more fine-grained and accurate representations. More importantly, we would like to see how the CNN-based and attention-based models can work together to improve downstream tasks such as classification.

In general, The main contributions of our work are as follows:

1. We proposed a feature fusion framework that combines CNN- and attention-based models. It delicately takes advantage of extracted features of multiple models, which can well overcome the bias when using only single-model representations.
2. We designed new structures for Vision Transformer (ViT) and Swin Transformer (Swin-T) that make Grad-CAM applicable in such attention-based backbones.
3. We reconstructed feature expressions based on three CAMs through an elaborated attention-selection mechanism. It is robust to data augmentation that may deviate the highlighted region in augmented images.

Overall experiment results on the IP102 dataset show that the reconstructed features get better classification performance, as well as outperform the widely-applied CNN-based models.

## Related work

### CNN-based backbone

**Figure 1:**
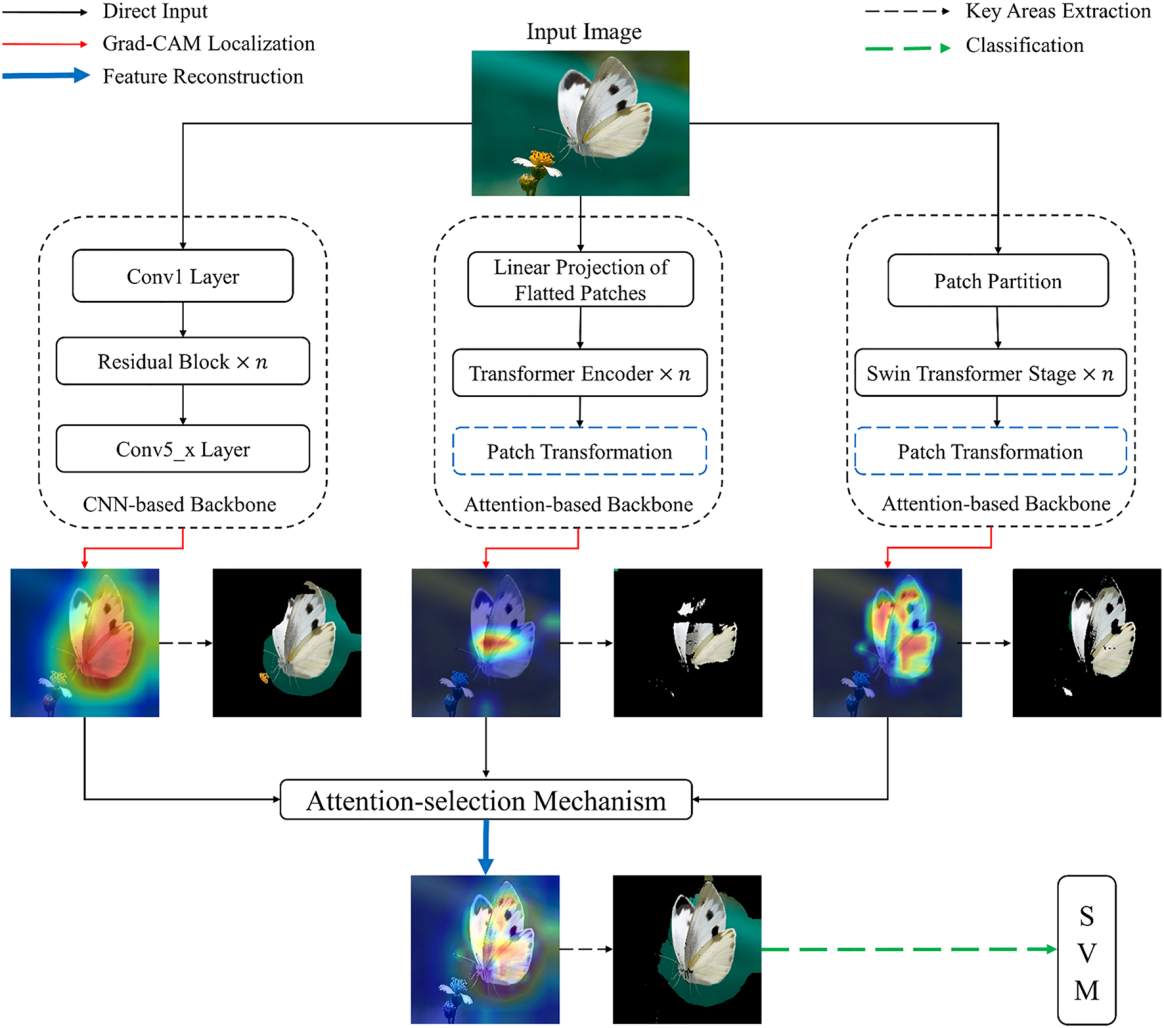
Overview of our method. We employ one CNN-based backbone (ResNet152) and two attention-based backbones (ViT in the middle, Swin-T on the right). The Patch Transformation Layers surrounded by a blue dotted line are the ones that we added to facilitate the generalization of Grad-CAM to attention-based architectures (details can be found in Fig. 2). The proposed attention-selection mechanism reconstructs the attention area into a new one (see Fig. 3). The different kinds of arrows represent different operations indicated by the upper legend in the figure.

The convolutional neural network is a widely applied and effective structure for hierarchical image feature extraction and representation. Among all the excellent CNN architectures, ResNet^29^ is a dominant one with excellent performance in various computer vision tasks^30^. It inserts shortcut connections that turn the network into its counterpart residual version, enabling these residual networks to be easier to optimize and gain accuracy from considerably increased depth. It is reported that ResNet152 gets the lowest top-1 and top-5 error rates both in 1-crop, 10-crop and single-model testing^29^. Therefore, we employed the representative ResNet152 as our CNN-based backbone to map the Grad-CAM. Besides, we found that ResNet152 always gave a continuous local region feature representation (see Figs. 1, 6, 7). It is noteworthy that many proceeding studies^3,11,31^ have employed Grad-CAM to test whether their CNN-based models focused on the correct region in insect images.

### Attention-based backbone

Compared with CNN-based models, Transformer is a solely attention-based network first proposed by Vaswani et al. for machine translation^28^. It has become the SOTA model in various natural language processing (NLP) tasks. Several attempts have been made to adapt Transformer from language to vision. Sparse Transformers^32^ employ scalable approximations to global self-attention to apply to images. A notable work is Vision Transformer^33^, which directly applies Transformer to the context of image processing. It interprets an image as a sequence of fixed-size patches and processes it by a standard Transformer encoder used in NLP. Besides, DeiT^34^ is an enhanced version of ViT that introduces several training strategies that make training on a small-scale dataset perform equally well. Similar to ViT, Swin Transformer^35^ splits an input RGB image into non-overlapping variable-size patches. It constructs hierarchical feature maps by merging image patches in deeper layers. Additionally, it adopts shifted window approach, which establishes correlation information among patches incorporated in different windows. It performs strongly on image classification, object detection, and semantic segmentation. In general, ViT and Swin-T respectively capture attention information in fixed image patches and variable windows; hence, the perceived features for an image are both unique and valuable. Therefore, we chose ViT and Swin-T as our two attention-based backbones to extract important features using Grad-CAM.

Unlike CNN structures, Grad-CAM has been seldomly applied to attention-based architectures since it is originally designed for CNN model families. In this study, we successfully generalized Grad-CAM to ViT and Swin-T backbones (details can be found in Section "Grad-CAM localization"), which may also shed light on the forthcoming attention models. Compared to the CNN-based model ResNet152, ViT and Swin-T pay more attention to more fine-grained global feature representations among image patches (see Fig. 1, 6, 7).

### Attention-selection backbone

As mentioned above, different kinds of vision backbones provide unique attention information when extracting features. It is necessary to fully consider and delicately select the highlighted regions as the reconstructed feature for further investigation. The methodology of the proposed attention-selection mechanism is similar to that of image fusion, which also refers to extracting and then combining the most meaningful information from different source images^36^. Moreover, image fusion essentially aims to make useful information more predominant, which is also one of the factors our attention-selection mechanism considers. Aided by a delicately-designed algorithm, the attention area can be well reconstructed based on different CAMs focused on distinct views.

Deep learning methods represent significant potential in image fusion since they are equipped with powerful abilities in feature extraction and data representation^37^. There are three kinds of main deep learning-based fusion methods: autoencoder (AE)-based^38^, conventional convolution neural network (CNN)-based^39–42^, and generative adversarial network (GAN)-based methods^43,44^. One of the CNN-based fusion methods employs elaborated loss functions and network structures to implement feature extraction, feature fusion and image reconstruction end-to-end, which shows robustness and good performance in various fusion tasks. A representative work is IFCNN^42^. IFCNN is a CNN-based general image fusion framework that demonstrates great generalization for fusing various images, such as multi-focus, infrared-visual, multi-modal medical and multi-exposure images. It is worth mentioning that this model has only been trained on the generated multi-focus image dataset^45^ (i.e. NYU-D2), while obtaining equal good performance on other fusion tasks for different types of images.

In this study, we employed IFCNN as the attention-selection backbone. The fine-grained features can be further reconstructed via a delicate attention-selection algorithm. A more detailed process of this attention-selection mechanism can be found in Section "Attention-selection mechanism".

## Methods

Figure 1 represents the overall framework of our method. Specifically, we first employed Grad-CAM to get attention CAMs by CNN-based backbone ResNet152 and attention-based backbones ViT and Swin-T. The highlighted region denotes the critical feature for the models to make decisions. The more important the region in the image is for predicting the concept, the more highlighted it will be reflected on the CAM. Then we reconstructed the attention area by the proposed attention-selection mechanism. This mechanism adopts IFCNN as a backbone, and an algorithm is designed to generate fine-grained features from the above CAMs. Specifically, we extracted features from the most valuable regions based on the attention information to reduce background noise interference. The proposed approach only requires local region information of the image, and we finally adopted an SVM classifier to handle the fine-grained features for classifying insects.

### Grad-CAM localization

As^16^ said, Grad-CAM applies to a wide range of CNN-based models and can visualize any activation in a deep network. Such important regions can be easily localized in the CNN-based model ResNet152. However, the visualization method should be different in ViT and Swin-T backbones due to the lack of the CNN layer.

**Figure 2:**
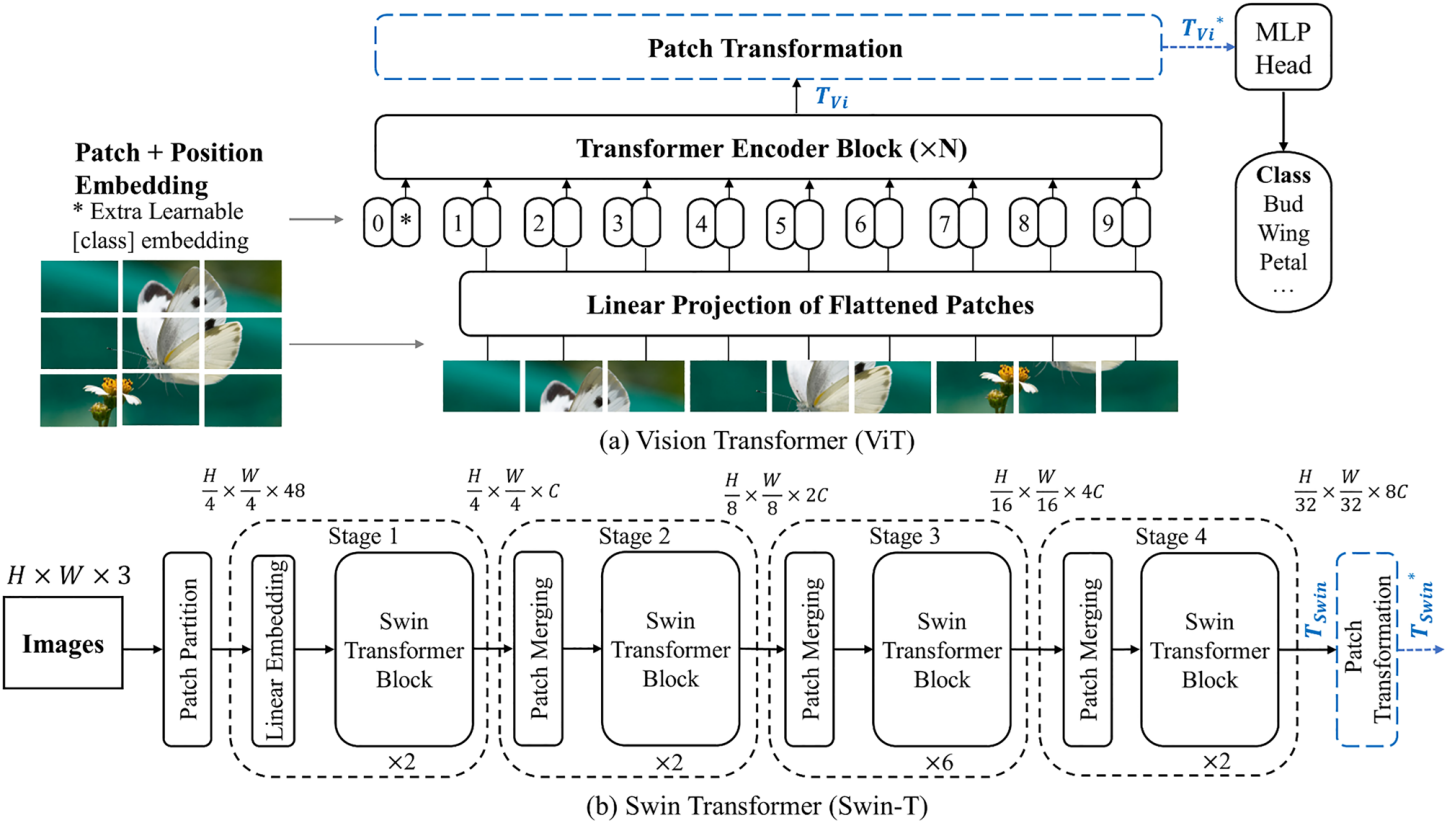
The modified architecture of ViT and Swin-T. A Patch Transformation Layer is both added to the last Transformer Block while the rest structures of the ViT and Swin-T remain unchanged. Note that ${{T}_{Vi}}^{*}$ and ${{T}_{Swin}}^{*}$ denote the reconstructed tensors, which are the ones we choose for following Grad-CAM localization work.

The standard Transformer requires 1D sequence embedding as the input. Therefore, both ViT and Swin-T reshape the 2D image $x\in {R}^{H\times W\times C}$ into a sequence of flattened 2D patches ${x}_{p}\in {R}^{N\times {(P}^{2}\times C)}$, where $(H,W)$ is the original resolution of that image, $C$ is the number of channels, ${P}^{2}$ is the size of each image patch, and $N=HW/{P}^{2}$ is the number of patches^33^. Typically, the output tensors ${T}_{Vi}$ and ${T}_{Swin}$ of the last Transformer Block (see Fig. 2) are in different formats, as shown in Eq. (1) and Eq. (2), respectively:

1 $$\begin{array}{c}{T}_{Vi}=\left(N+\left[class\right]token\right)\times \left({P}^{2}\times C\right)\end{array}$$

where $P\times P$ is the resolution of each image patch in ViT, $[class]token$ is a trainable parameter uniquely designed for classification.

2 $$\begin{array}{*{20}c} {T_{Swin} = \underbrace {{\left( {\frac{H}{32} \times \frac{W}{32}} \right)}}_{M \times M} \times \left( {8C} \right)} \\ \end{array}$$

where $M\times M$ denotes the window size.

To make the localization results comparable and convincing, as well as make Grad-CAM generalize to the attention-based architecture, we added a Patch Transformation Layer to reshape the above output tensors ${T}_{Vi}$ and ${T}_{Swin}$. That is how the ${{T}_{Vi}}^{*}$ and ${{T}_{Swin}}^{*}$ come from, and the two tensors are used to calculate the classification importance degree.

Additionally, we can see that both the outputs after the Patch Transformation Layer in two Transformers hold a similar format as $(K)\times ({C}^{*})$. Then we can delicately treat the output tensors as a $\sqrt{K}\times \sqrt{K}$ spatial image with ${C}^{*}$ channels. Note that in ViT we should replace $K$ with $K-1$ since the $\left[class\right]token$ is included. In doing so, the following Grad-CAM visualization work for CNN-based and attention-based models is agreed.

### Key area features extraction

#### Single-attention information

For the attention information toward a specific model, key areas could be extracted on the basis of important regions that denote important decision-making features with underlying numerical explanations in mapped CAM. Essentially, the mapping process of Grad-CAM calculated the neuron importance weight of every pixel in the final layer or block output. Therefore, one feasible way to extract the most important region is to retain pixels according to these numerical weights. We set a threshold for neuron importance weights and only the pixels beyond the threshold would be kept, while the remaining pixels would be masked to black.

#### Attention-selection mechanism

In this study, we proposed an attention-selection mechanism to fully employ unique attention information from different views. In other words, we reconstructed the highlighted regions based on distinct CAMs to get more comprehensive and fine-grained attention areas. Specifically, we adopted IFCNN^42^, a general framework for image fusion, as a backbone to extract attention information. Furthermore, we modified the CNN structure of IFCNN and added an elaborately-designed attention-selection algorithm to reconstruct the attention area.

**Figure 3:**
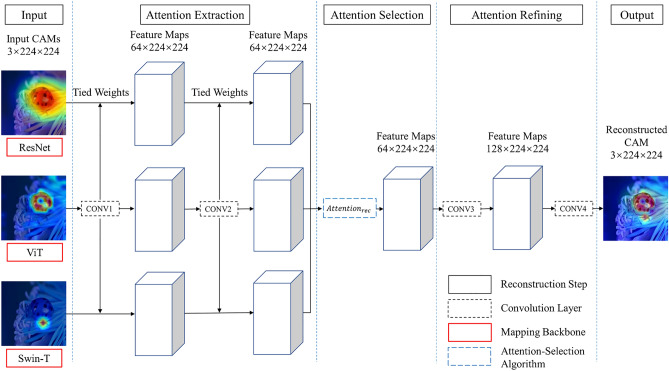
The implementation detail of our proposed attention-selection mechanism. The framework is similar to IFCNN, while we modified its CNN structure and added an attention-selection algorithm. The exact processing meanings denoted by the different rectangles are illustrated in the legend at the bottom right.

Overall, there are four convolutional layers in the IFCNN backbone (see Fig. 3). The first two are used to extract the attention features of the input CAMs gradually. After the process of attention-selection, the third CNN plays the role of tuning the convolutional features. Compared to the original IFCNN structure, we modified the output channels of CONV3 to refine the attention information. The CONV4 is adopted to reconstruct these deep feature maps into the 3-channel output. Here we fix the number of input images $N = 3$ since we have three different CAMs generated by ResNet152, ViT and Swin-T backbones.

The attention-selection mechanism aims to better extract the most important region in original CAMs. As mentioned above, a more highlighted region in the CAM represents a higher importance degree for predicting the concept. Unlike extracting the most valuable features from a single model in which we directly adopted the neuron importance weights, this time we assigned each pixel a highlighted weight ${w}_{h}$ according to the degree of highlighted regions. Most importantly, these pixel-wise weights are numerically equal to neuron importance weights in Grad-CAM. After the process of the first two convolutional layers, these so-called neuron importance weights would be retained in feature maps as ${\alpha }_{i}^{k}$. The detailed attention-selection operation is shown as follows:

3 $$\begin{array}{c}{\widehat{f}}^{k}\left(x,y\right)=\underset{i}{{\mathit{Attention}}_{\mathit{rec}}}\left({f}_{i, C2}^{k}\left(x,y\right)\right), 1\le i\le N\end{array}$$

where ${f}_{i, C2}^{k}$ denotes the $k$ th feature map of the $i$ th input image extracted by CONV2, ${\widehat{f}}^{k}$ denotes the $k$ th channel of feature maps after attention-selection, and $\mathop {Attention_{rec} }\limits_{i}$ refers to the selection algorithm.

4 $$\begin{array}{*{20}c} {Attention_{rec} = Eigen\left\{ {w_{0} \times \bigcap\limits_{i = 1}^{3} {\alpha_{i}^{k} } + w_{1} \times \left( {\bigcup\limits_{i = 1}^{3} {\alpha_{i}^{k} } - \bigcap\limits_{i = 1}^{3} {\alpha_{i}^{k} } } \right)} \right\}} \\ \end{array}$$

where ${\alpha }_{i}^{k}$ represents the neuron importance weights in Grad-CAM of ${f}_{i, C2}^{k}$, ${w}_{0}$ and ${w}_{1}$ are balanced coefficients used to increase the weights of the most important regions while reducing the ones of less important areas, $Eigen$ denotes a smoothing method^46^ to minimize noises.

The overall approach to extract key area features for single and multiple attention information is presented in Algorithm 1 (Note that the original image represents the one before Grad-CAM localization). More importantly, our attention-selection mechanism is robust to augmented images where the reconstructed CAMs can well eliminate the deviation caused by different augmentations. It also combines the most judgmental features of different models. Details can be seen in Section "Data Augmentation".
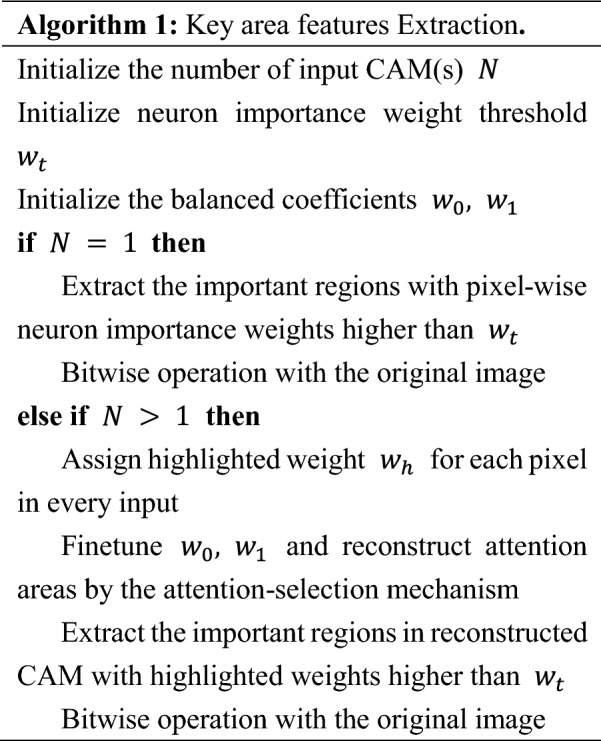


Overall, these elaborated features, especially the reconstructed ones, aim to better depict the integral pattern of insects and further reduce the background noise as much as possible, especially when the insects and the background share the similar color.

### Experiments

We respectively evaluated the classification performance using single-attention information based on ResNet152, ViT and Swin-T backbones. Furthermore, we reconstruct the attention area via our proposed attention-selection mechanism. All the above three backbones are pre-trained on ImageNet^47^. The Grad-CAM localization and attention-selection process are based on the PyTorch framework^48^. We adopted the SVM classifier in Scikit-learn^49^ to train and test the insect recognition performance.

### Dataset

**Figure 4:**
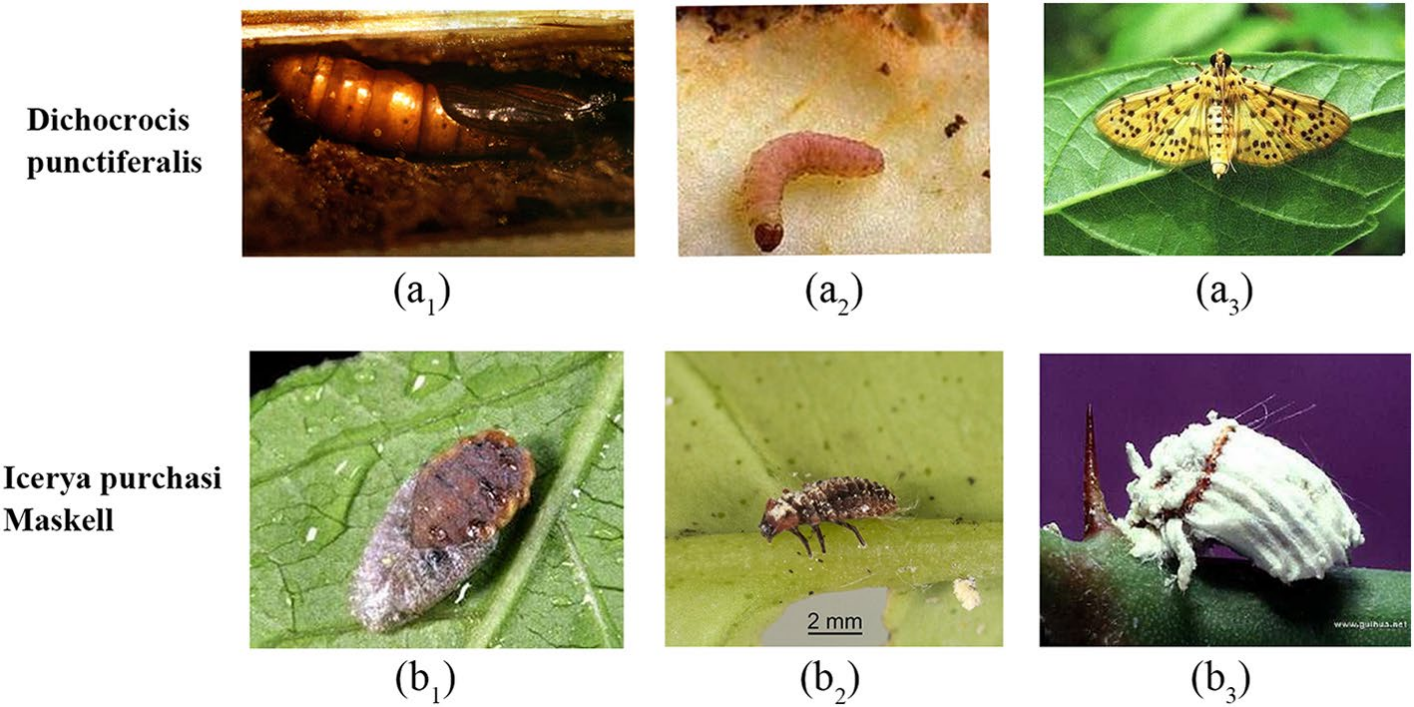
Examples of different growth forms of the same category in IP102 where a_1_ ~ a_3_ and b_1_ ~ b_3_ denote the pupa, larva, and adult of Dichocrocis punctiferalis and Icerya purchasi Maskell, respectively.

**Figure 5:**
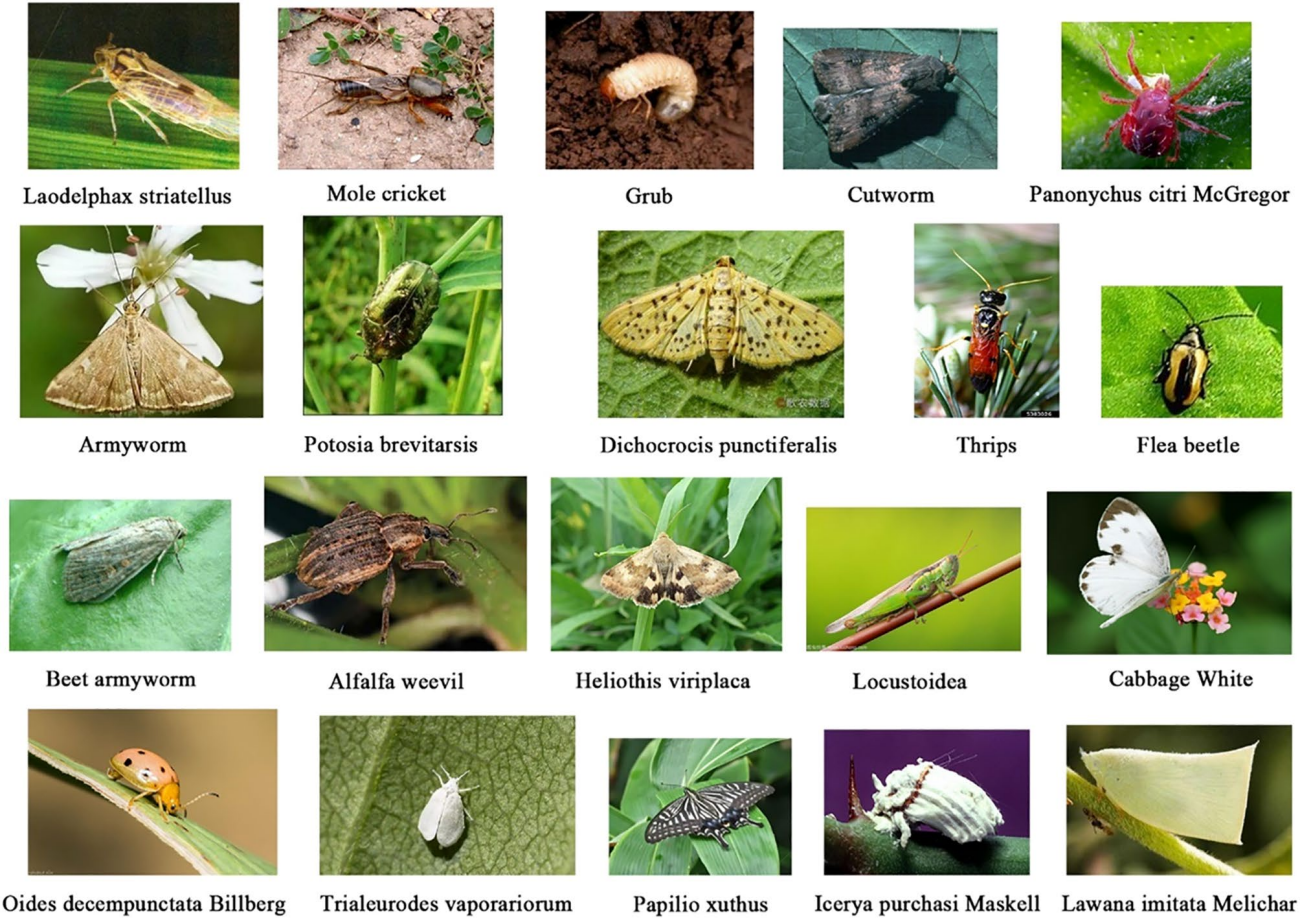
The randomly selected 20 kinds of adult insect pests from the IP102 dataset.

The IP102 dataset contains more than 75 000 images belonging to 102 categories^9^. The main difference between IP102 and other datasets is that it captures different growth conditions of one specific pest in the same category (e.g., pupa, larvae and imago). More importantly, some kinds of insects share similar appearances in non-adult conditions, increasing the difficulty of correctly identifying these insects. Examples of insect images in different growth stages can be found in Fig. 4. We tested the classification performance on randomly extracted 20 insect species (see Fig. 5) and the whole IP102 dataset. Detailed experiment results can be found in Section "Classification Results".

The classification and detection results of the IP102 dataset have proved it to be a challenging pests dataset^9^. Except for the overall quantity imbalance in different categories, the number of different growth forms that belong to the same category equally varies. Specifically, the adult insects get far more images than other growth types in numbers, increasing the generalization difficulty of subsequent classifiers. Therefore, we implemented data augmentation to offset such quantity variance. Moreover, owing to the good performance of handling few-shot samples, the SVM classifier is used to mitigate the scarcity of specific growth stage images.

### Data augmentation

We randomly adopted seven augmentation methods^50^ to offset the intra-class and inter-species quantity variance (see Table 1). Note that an image can use more than one data augmentation method.

**Table 1:** Different augmentations. HF: Horizontal Flip; BC: Brightness Contrast; GB: Gaussian Blur.

Methods	Limit	Probability
HF	–	0.5
BC	Brightness:(− 0.3, 0.3) Contrast:(− 0.3, 0.3)	0.4
Shift	(− 0.65, 0.65)	0.4
Scale	(− 0.1, 0.1)	0.3
Rotate	(-45, 45)	0.4
GB	(3, 7)	0.5
Sharpen	Alpha:(0.2, 0.5) Lightness:(0.5, 1.0)	0.3

**Figure 6:**
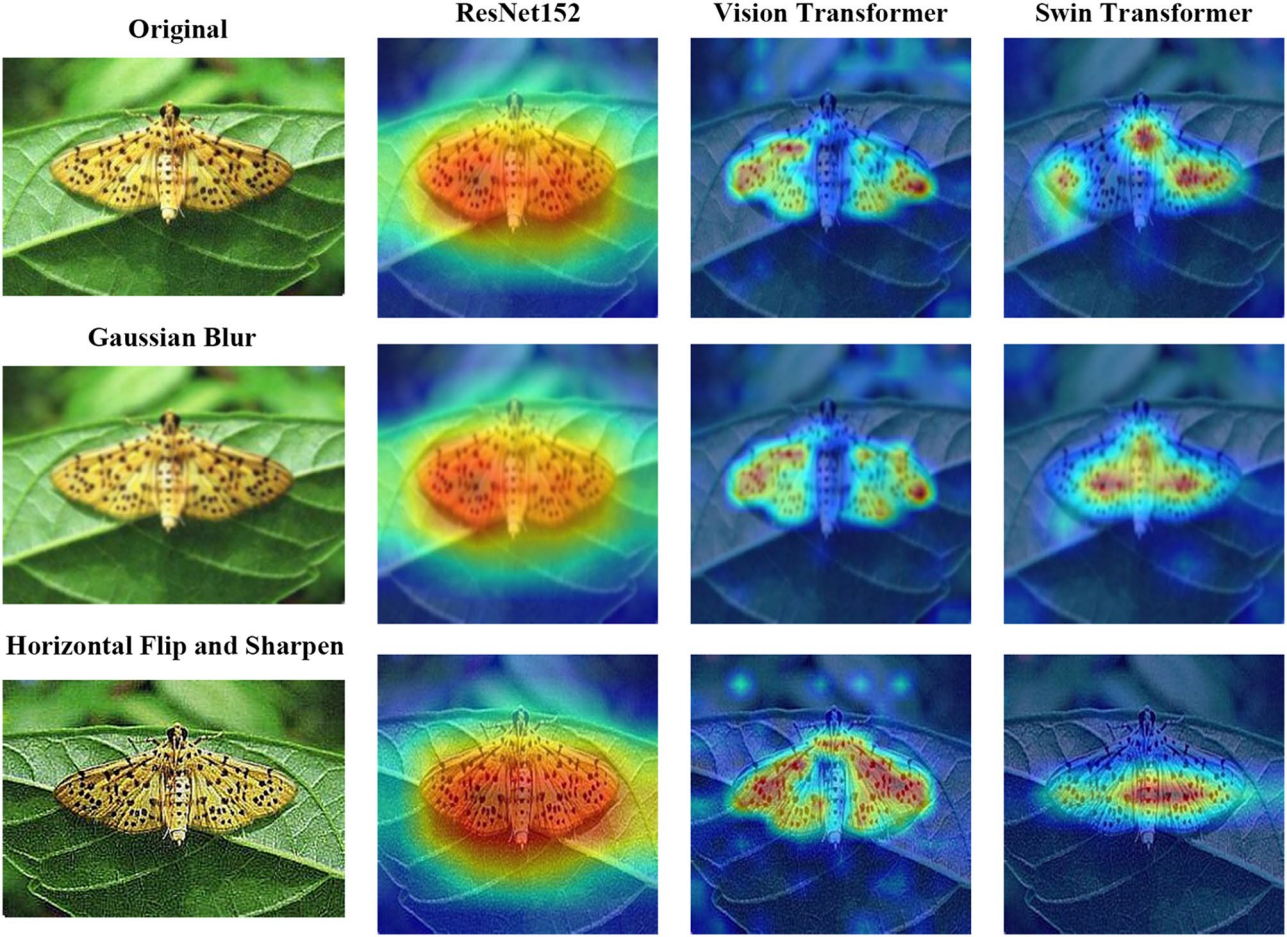
Examples of the depicted CAMs of the original and augmented images. Different augmentations may cause the deviation of important regions localized by the three backbones to varying degrees.

In some cases, different data augmentation methods may deviate the highlighted portions in CAMs among these three backbones. Figure 6 gives detailed examples. We can infer that different augmentations greatly changed the attention information for the same image.

**Figure 7:**
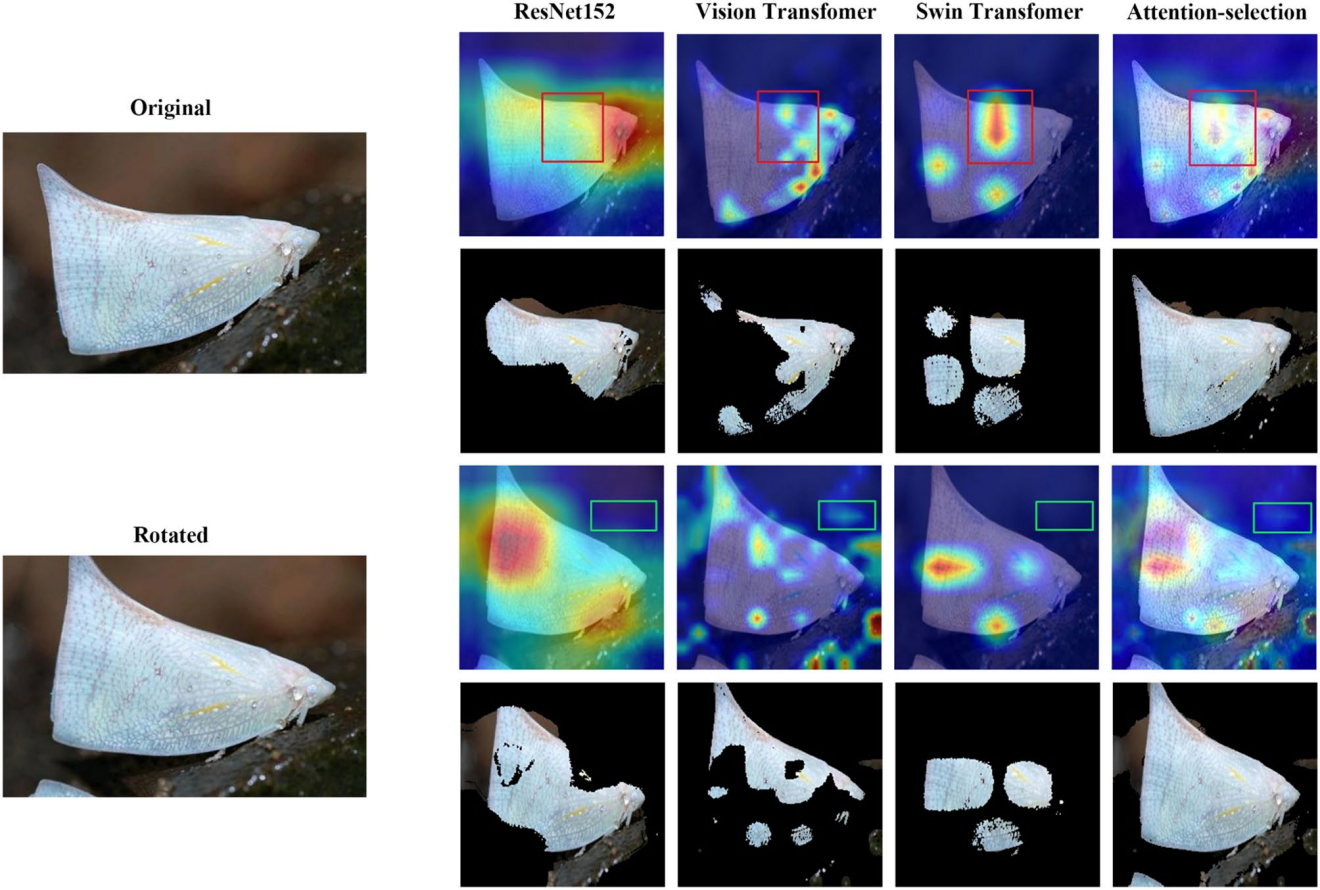
Overall feature representation for a pair of original and rotated pest images. The first and third rows are three CAMs and the feature reconstruction results. The second and fourth rows are the corresponding key regions, which are the input features of the SVM classifier. This example shows that our proposed attention selection mechanism can effectively reduce disturbing feature regions (green boxes) and strengthen key feature regions (red boxes).

However, the attention area reconstructed by the attention-selection mechanism can well eliminate these influences among different augmented images (see Fig. 7), which shows that the proposed feature reconstruction scheme significantly improves the stability of key feature extraction.

### Attention-selection Analysis

In this part, we quantitatively and qualitatively evaluate the proposed attention-selection mechanism (see Table 2 and Fig. 7, respectively). Similar to the principle of image fusion, the selection of attention information should integrate as many commonly recognized features as possible from each source image into the feature images, which makes sharing the same evaluation metric possible. We employed spatial frequency (SF)^51^ and average gradient (AG)^52^, which measure the textural information amount of the fusion image from two different statistical views^42^ to evaluate the performance of the attention-selection mechanism quantitatively. Furthermore, Fig. 7 qualitatively presents a holistic view of features extracted from different backbones and demonstrates good robustness of reconstructed features to data augmentation.

**Table 2:** Comparison of quantitative results on the whole IP102 dataset. The higher number represents better fusion performance.

Metrics	IFCNN	Attention-selection
SF	0.028	0.036
AG	17.871	19.452

By modifying the basic CNN structure and fusion scheme in IFCNN architecture, we can see that our proposed attention-selection mechanism improves feature fusion performance in Table 2.

### Classification results

We first tested the classification performance using single-attention information on randomly selected 20 insect species and the whole IP102 dataset. Afterward, we reconstructed the attention area to obtain more fine-grained attention information and then evaluated the recognition effect based on the same two sets of insects. The evaluation metrics include F1-score (denoted as F1), recall (denoted as Rec), precision (denoted as Pre), and accuracy (denoted as Acc).

Tables 3 and 4 reported the detailed classification results on 20 randomly selected insect species and 102 insect categories from the IP102 dataset, respectively. We can see that our attention-selection-based model significantly improves the overall classification performance.

**Table 3:** Classification performance on 20 randomly selected insect species from the IP102 dataset. Note that ‘Method’ refers to the network framework for extracting features.

Methods	F1	Rec	Pre	Acc
ResNet152 backbone	53.1	52.6	53.7	48.5
ViT backbone	46.8	47.0	46.7	50.5
Swin-T backbone	50.8	49.5	52.2	46.2
Ours	**61.3**	**59.0**	**63.8**	**62.1**

Significant values are in bold.

**Table 4:** Classification performance on the whole 102 insect species from the IP102 dataset. Note that ‘Method’ refers to the network framework for extracting features.

Methods	F1	Rec	Pre	Acc
ResNet152 backbone	51.4	49.0	54.0	46.3
ViT backbone	48.4	47.5	49.4	52.3
Swin-T backbone	49.1	47.2	51.2	48.0
Ours	**60.3**	**59.7**	**60.9**	**65.6**

Significant values are in bold.

Furthermore, we compared the classification results of the reconstructed features with those of other well-known CNN structures that have been proven effective on the whole IP102 dataset (see Table 5).

**Table 5:** Comparison of the classification performance of CNN feature-based model with our feature reconstruction model.

Methods	F1	Acc
AlexNet^53^	34.1	41.8
GoogleNet^54^	32.7	43.5
VGGNet^55^	38.7	48.2
ResNet^29^	40.1	49.4
FR-ResNet^11^	54.1	55.2
FusionSum^6^	-	61.9
Ours	**60.3**	**65.6**

Significant values are in bold.

Compared to other widely applied CNN feature-based models, we can see from Table 5 that our proposed attention-selection-based approach shows significant advantages. The difference between their models and our approach is twofold. Instead of using the entire image features, we only used part of the image information that represents the most critical features. In addition, fully connected layers are usually used for classification in various CNN-based models. However, considering the imbalance in different growth forms, we adopted SVM as the classifier to handle limited insects in specific stages.

Moreover, we compared the training time with the most advanced models in Table 6. Note that our training and test process was only implemented on the SVM classifier, which employed key area features to recognize insects. Despite the competitive recognition results, our approach requires less training time that can quickly be applied to the test set.

**Table 6:** Comparison of the state-of-the-art models implemented on the IP102 dataset.

Methods	Training time (h)
GAEnsemble^10^	3.2
Inception-v3^8^	7.8
Ensembles^56^	4.5
Ours	**1.8**

Significant values are in bold.

### Ablation study

To check which model provides the most critical feature representations, we evaluated the classification performance of the proposed method when only two backbone features were reconstructed.

Table 7 shows a detailed comparison of classification performance. The first two combinations that incorporate RseNet152 obtained better performance than the selection of two vision transformer backbones. As described in Sect. "CNN-based backbone", ResNet152 introduces a wider range of local feature responses as much as possible, making the highlighted regions in CAMs more continuous. Whereas in ViT and Swin-T, the extracted feature representations are fine-grained and decentralized, as they focus more on global information associations. The large organizational differences between the two Transformers will bring more distinct but valuable information. Overall, only the fusion of the CNN-based and attention-based backbones can comprehensively take advantage of local and global features, which is greatly helpful in recognizing specific insects.

**Table 7:** Classification performance on the whole 102 insect species of the IP102 dataset. ‘Backbones’ represent which two models are combined for feature reconstruction. Note that the last row corresponds to the result of considering all three backbone features.

Backbones	F1	Rec	Pre	Acc
ResNet152 + ViT	55.3	53.7	57.1	56.8
ResNet152 + Swin-T	56.0	53.6	58.7	57.2
ViT + Swin-T	52.7	51.3	54.2	53.3
ResNet152 + ViT + Swin-T	**60.3**	**59.7**	**60.9**	**65.6**

Significant values are in bold.

## Discussion

Experiments on IP102 showed that our attention-selection features outperformed many widely used CNN-based models in terms of F1-score, recall, precision, and accuracy in insect classification. The reduced performance in single-model features also validates the effectiveness and necessity of our fuse schemes. Additionally, our approach is competitive with state-of-the-art models. Except that less training time is needed, the SVM is also a lighter classifier that does not require numerous training parameters. The adopted IP102 is a highly unbalanced dataset^1^, in which the number of samples in each class and the images of different growth forms largely vary. To mitigate the intra-class unbalance, we implemented data augmentation to offset the quantity variance in different classes. Moreover, the SVM classifier was adopted to learn from limited images in specific growth stages to alleviate inter-species unbalance. Finally, we demonstrated that our reconstructed features could better reinforce key insect features and attenuate background interference as much as possible. The proposed attention-selection mechanism is also robust to data augmentation that may deviate the important regions in CAM.

## Conclusion

Accurately recognizing insect pests has always been critical and meaningful for improving agricultural products and the ecological environment. As CNN-based models have become a widely-applied and well-performed tool in the vision field, many researchers focused on structure modification and ensemble ways to better recognize insect images. Since attention-based models are seldom used in this area, and few studies consider both the advantages of CNN and vision Transformers in feature extraction, here we proposed a feature fusion framework to enable more fine-grained feature representations. We first employed one CNN-based and two attention-based backbones to localize the important regions in insect images by Grad-CAM. During this process, we successfully made Grad-CAM applicable to attention-based architectures ViT and Swin-T. Afterward, we reconstructed attention areas based on the Grad-CAM by a delicately-designed attention-selection mechanism. The proposed approach obtained strong classification performance compared to single-attention features and other widely-applied CNN-based models on the IP102 dataset. It is worth mentioning that we only adopted key region features, which were extracted according to pixel-wise weights, as the input of the SVM classifier. Most importantly, our proposed attention-selection mechanism could reconstruct features from broader CAMs, which are not limited to extracting attention in insect images but could be generalized to other samples in different fields like animals, objects, plants, etc. Future research could be done when different objects are included (i.e., more than two kinds of insect pests shown in the same image). We believe this study can inspire future developments in comprehensively taking advantage of multiple model features, which may further improve insect recognition performance.

## Data Availability

The IP102 dataset used in this study is available at: https://github.com/xpwu95/IP102.
